# Multifunctional Drugs-Loaded Carbomol Hydrogel Promotes Diabetic Wound Healing via Antimicrobial and Immunoregulation

**DOI:** 10.3390/gels9090761

**Published:** 2023-09-18

**Authors:** Hehui Wang, Jiale Jin, Chi Zhang, Fangyi Gong, Baiwen Hu, Xiaochuan Wu, Ming Guan, Dongdong Xia

**Affiliations:** 1 Department of Orthopedics, The First Affiliated Hospital of Ningbo University, Ningbo 315000, China; fyywanghehui@nbu.edu.cn (H.W.); fyyzhangchi@nbu.edu.cn (C.Z.); fyygongfangyi@nbu.edu.cn (F.G.); fyyhubaiwen@nbu.edu.cn (B.H.); fyywuxiaochuan@nbu.edu.cn (X.W.); 2 Department of Orthopedics, The First Affiliated Hospital, Zhejiang University School of Medicine, Hangzhou 310000, China; 22018143@zju.edu.cn

**Keywords:** diabetic wound healing, carbomer, antimicrobial, immunoregulation

## Abstract

Diabetic wound healing poses a significant clinical dilemma. Bacterial infection and immune dysregulation are the predominant reasons. However, conventional wound dressings with a single treatment approach often limit therapeutic efficacy and continue working with difficulty. These limitations cause high treatment failure for diabetic wounds. In this study, we developed a multiple drug-loaded carbomer hydrogel containing Que/Van/Rif (QVR-CBMG) for the simultaneous treatment of infection and immune dysregulation. Honeycomb-like QVR-CBMG hydrogel exhibits excellent abilities to eliminate bacterial infection and biofilms in vitro. Moreover, QVR-CBMG hydrogel possesses an immunomodulatory capacity via affecting the Sirt3/SOD2 signaling pathway to promote M2 macrophages. Furthermore, QVR-CBMG hydrogel effectively promotes wound healing in diabetic rats through several mechanisms. The multidrug-loaded wound dressing not only eliminates bacterial infection and facilitated angiogenesis but also promotes collagen deposition and remodulates the local immune microenvironment in the areas of wounds. In summary, this synthetic strategy to eliminate infection and regulate immune disorders has potential translational value for the prevention and management of diabetic wounds.

## 1. Introduction

The management of diabetic wounds presents a significant clinical challenge due to their difficulty in healing and the potential for severe complications, leading to substantial economic and societal burdens [1]. The microenvironment of a diabetic wound is typically characterized by hyperglycemia, impaired circulation, and dysfunctional immune responses, which create a favorable environment for bacterial colonization and proliferation [2,3]. Worse, biofilms, a highly organized bacterial community, are prone to forming in diabetic wounds and resist clearance [4]. Additionally, biofilm infections prolong the inflammatory response and ultimately result in delayed or non-healing wounds [5]. Traditional treatment approaches require multistep debris and prolonged antibiotic administration; failure, however, remains high [6]. A novel therapeutic strategy to simultaneously address biofilm infection and chronic inflammatory microenvironments is therefore clinically needed in diabetic wound care.

Appropriate antibiotic therapy remains the most common choice to effectively control infection [7]. Rifampicin (Rif), a broad-spectrum fluoroquinolone antibiotic, possesses the capability to penetrate biofilms and exhibit antibacterial effects [8]. However, high dosages are required, which, in turn, increases the cytotoxicity [9]. In recent years, antibiotic combinations have emerged as a strategy to combat biofilms [10]. A combination of Rif and vancomycin (Van) has shown reciprocal reinforcement of biofilm clearance, resting on their unique antibacterial mechanisms [11]. However, their role in infected diabetic wound care is poorly understood.

In addition to addressing bacterial infections, the dysfunctional immune microenvironment in diabetic wounds is of equal concern [12]. The secretion of chemokines or cytokines by local macrophages in diabetic wounds leads to the over-recruitment of monocytes, which later shift to the M1 inflammatory phenotype [13]. These imbalanced immune responses result in a chronic inflammatory cycle in the wound. The prolonged imbalance between M1 and M2 macrophage phenotypes leads to the development of non-healing wound patterns [14]. Quercetin (Que), as reported, has been used to treat various inflammatory diseases with its roles in anti-inflammatory properties [15]. However, the poor water solubility limits the bioavailability of Que [16].

Hydrogels have been widely used in wound healing [17,18]. Another advantage of using hydrogels is their ability to protect drugs or compounds from degradation, thereby prolonging their release time and potency [19]. Among different types of hydrogels, carbomer hydrogel has gained popularity as an ideal carrier for treating diabetic wounds due to its excellent moisturizing properties and ease of use and management [20].

**Scheme 1:**
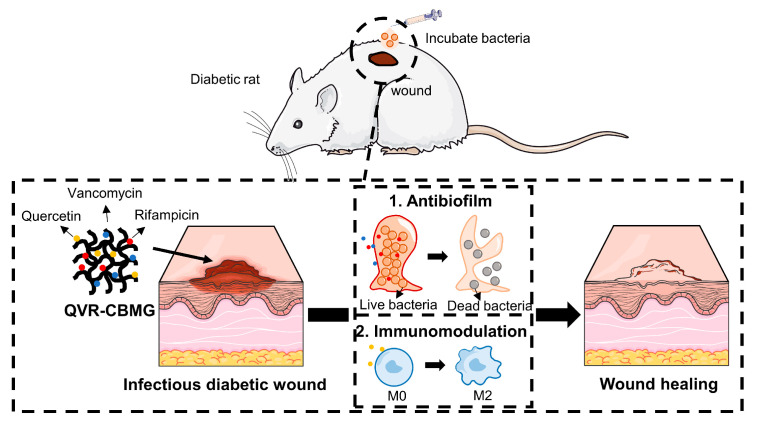
Schematic illustration of biofilm elimination and immunomodulation by QVR-CBMG hydrogel for infected diabetic wound healing. CBMG: Carbomer. Rif: Rifampicin. VR: Vancomycin-Rifampicin. QVR: Quercetin-Vancomycin-Rifampicin.

Therefore, we developed a carbomer hydrogel (CBMG) loaded with Rif/Van/Que (QVR-CBMG) to treat diabetic-infected wounds. We first evaluated its antibacterial effects against both planktonic bacteria and biofilms in vitro. Then, we investigated the influence of this hydrogel on macrophage polarization and its potential mechanisms. A diabetic-infected wound model was performed to evaluate the antibacterial and immunomodulatory properties of QVR-CBMG to promote wound healing in vivo (Scheme 1). Our study aims to demonstrate whether QVR-CBMG could serve as an effective and safe approach to accelerate diabetic-infected wound repair and identify its underlying mechanisms.

## 2. Results and Discussion

### 2.1. Synthesis and Characterization of Hydrogels

**Figure 1:**
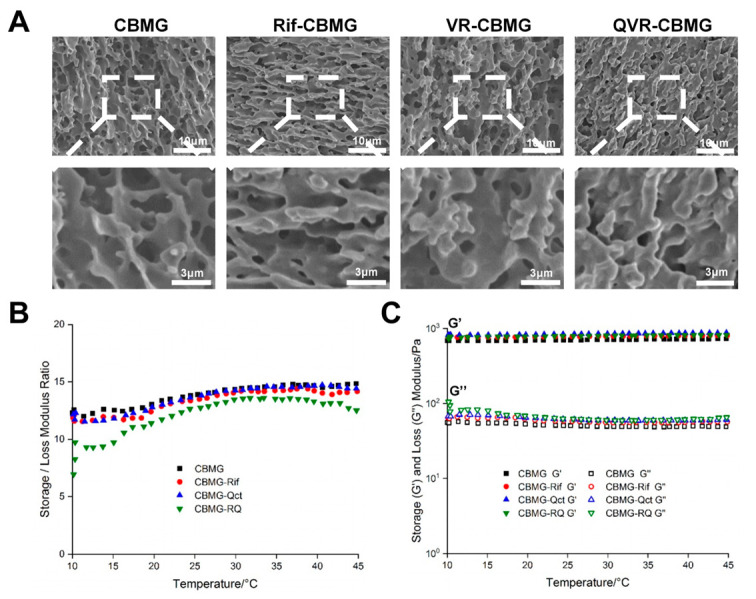
(**A**) Representative SEM micrographs of CBMG loaded with various drugs. (**B**) The storage/loss modulus ratio of the hydrogels were measured using a rotary rheometer. (**C**) The elastic modulus G′ and storage modulus G″ of the hydrogels were measured using a rotary rheometer.

The microstructures of the hydrogels were examined using SEM. Figure 1A illustrates that CBMG, Rif-CBMG, VR-CBMG, and QVR-CBMG hydrogels exhibited similar honeycomb-like microstructures. In addition, there was a decrease in porosity and restricted pore size for VR-CBMG and QVR-CBMG, which was related to the interactions between the drugs and the hydrogel matrix. These interactions, including hydrogen bonding, electrostatic interactions, or hydrophobic interactions, potentially alter the structure and arrangement of the hydrogel network, causing pore rearrangement and size reduction [21,22,23]. Further investigation using advanced imaging techniques and characterization methods is needed to fully understand the mechanisms underlying the observed pore size reduction. Moreover, lower pore sizes in hydrogels tend to correlate with improved structural stability and increased crosslinking density. This not only contributes to the longevity of wound treatment but also enhances the overall performance of the hydrogel in delivering therapeutic agents [24]. Additionally, the viscoelastic behavior and functional properties of the hydrogels, including their cushioning and energy absorption capabilities, are influenced by the elastic modulus and loss modulus. The rheological measurements revealed that the drug content had less effect on the modulus of the carbomer-based hydrogel (Figure 1B,C). This indicates that QVR-CBMG hydrogel possesses mechanical properties comparable to other hydrogels. 

### 2.2. Cell Biocompatibility and Antibacterial/Antibiofilm Activities of Hydrogels

The biocompatibility of hydrogels was assessed using the CCK-8 assay. CCK-8 analysis showed that the hydrogels had no effect on BMDMs at any time point (Figure S1). The excellent biocompatibility of the carbomer hydrogel has been a key factor in its widespread application [25,26]. Single-use MRSA infections pose a great challenge to treating diabetic wounds [7]. According to the turbidity of the bacterial culture medium with different concentrations of Van-CBMG, we found that Van-CBMG (4 μg/g) was the minimum inhibitory concentration for MRSA. However, a marginal effect on the biofilm was observed at this concentration. When combined with Rif, the biofilm removal effect can be increased in a Rif concentration-dependent manner. Strikingly, crystal violet-stained biofilm was almost eradicated when combined with Rif at 64 μg/g. Therefore, we used VR-CBMG with this content of Van (4 μg/g) and Rif (64 μg/g) for further biological experiments (Figure S2). 

**Figure 2:**
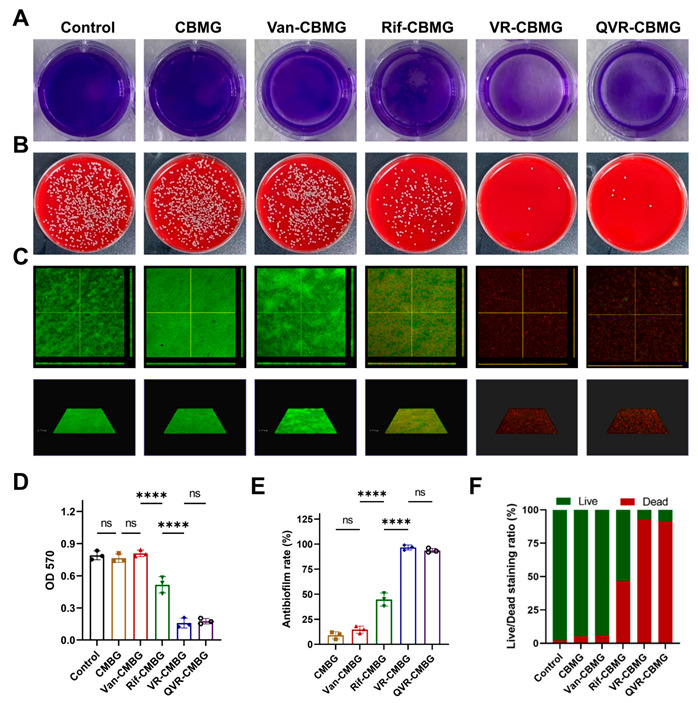
(**A**) A 24-h-old biofilm in each group was stained with 1% crystal violet. (**B**) A 24-h-old biofilm in each group was detached and spread on sheep blood agar using the spread plate method. (**C**) Bacterial live/dead staining for bacteria in a biofilm, where live cells stained with STYO 9 showed green and dead cells stained with PI showed red. (**D**) Corresponding analysis of the absorbance of a crystal violet-de-stained biofilm at 570 nm. (**E**) The antibiofilm rate was measured based on CFU counting. (**F**) The proportion of live and dead bacteria in the biofilm according to the fluorescence intensity of STYO9 and PI. Data represent the mean ± SD; n = 3; ns: no significant; **** *p* < 0.0001.

We then conducted an in vitro evaluation of the MRSA biofilm-clearing capabilities of QVR-CBMG hydrogel. Initially, we utilized 1% crystal violet staining to demonstrate the potential of the hydrogel for clearing biofilms. The hydrogel loaded with Rif showed superior efficacy in biofilm clearing compared to the hydrogel loaded with Van. However, the QVR-CBMG and VR-CBMG hydrogels exhibited the most effective biofilm clearance, as confirmed by IF stains (Figure 2A,C). Additionally, the agar diffusion assay indicated that the combination therapy with QVR-CBMG hydrogel displayed higher antimicrobial potency than using Van or Rif alone. Notably, the addition of Que did not compromise the antimicrobial and biofilm-clearing abilities of the hydrogels (Figure 2B). The corresponding statistical results also supported this observation (Figure 2D–F). There results showed that QVR-CBMG maintained the excellent antibiofilm efficacy of the Rif and Van combination, which may serve as a potential anti-infective wound dressing. 

### 2.3. QVR-CBMG Hydrogel Affected the Polarization of Macrophages

**Figure 3:**
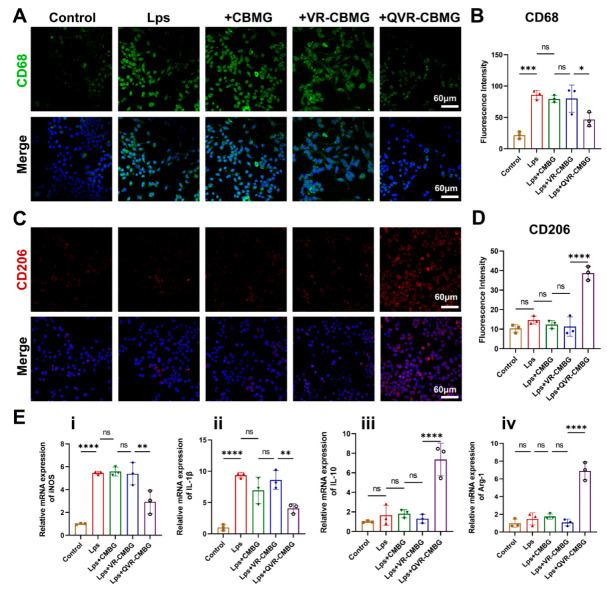
(**A**) Representative IF images of CD68 with different treatments in BMDMs. (**B**) Quantification analysis for CD68. (**C**) Representative IF images of CD206 with different treatments in BMDMs. (**D**) Quantification analysis for CD206. (**E**) q-PCR assay of the gene expression of proinflammatory genes ((i) iNOS and (ii) IL-1β) and anti-inflammatory genes ((iii) IL-10 and (iv) Arg-1). Data represent the mean ± SD; n = 3; ns: no significant; * *p* < 0.05, ** *p* < 0.01, *** *p* < 0.001, and **** *p* < 0.0001.

The dysfunctional immune microenvironment is a significant obstacle to the repair of diabetic wounds [14]. Numerous therapeutic approaches have been developed to improve the deterioration of the wound microenvironment [27,28,29]. Que, a widely researched and applied drug, has been shown to effectively improve the malignant polarization of macrophages [30]. Que was therefore introduced to VR-CBMG to regulate the macrophage polarization. Then, we investigated the potential protective effects of our hydrogel on macrophage polarization under lipopolysaccharide (LPS) stimulation. According to previous studies, CD68/CD206 are markers of M1/M2 macrophages [31]. Coculturing macrophages with the QVR-CBMG hydrogel led to a reduction in CD68 expression and an increase in CD206 expression, while CBMG and VR-CBMG did not seem to influence macrophage polarization (Figure 3A,C). The corresponding statistical results also supported that QVR-CBMG can effectively switch the M1 macrophage to the M2 macrophage (Figure 3B,D). Additionally, qPCR was used to evaluate the impact of the hydrogel on the expression of macrophage polarization-related genes. iNOS and IL-1β are indicators of M1 polarization, whereas IL-10 and Arg-1 are associated with M2 polarization. The expression of related genes demonstrated that QVR-CBMG effectively downregulated M1 polarization-related gene expression and upregulated M2 polarization-related gene expression, consistent with the fluorescence results (Figure 3E). Taken together, the results demonstrate that the QVR-CBMG hydrogel can effectively reverse the LPS-induced M1 polarization of macrophages.

### 2.4. QVR-CBMG Hydrogel Activated the Sirtuin 3(SIRT3)/Superoxide Dismutase (SOD2) Pathway

**Figure 4:**
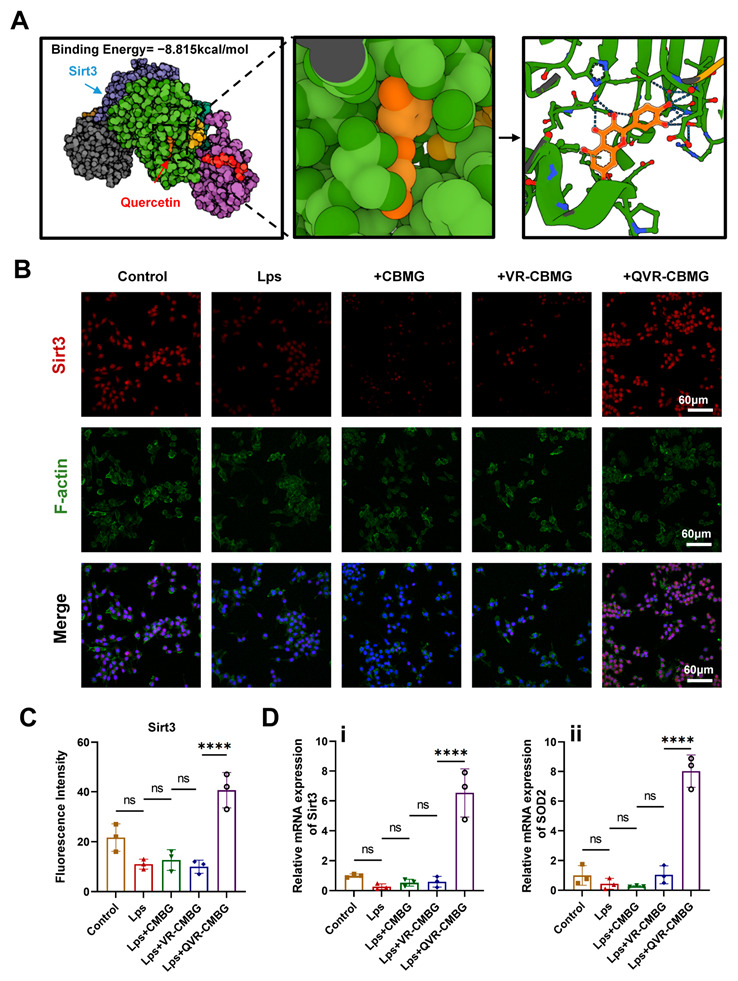
(**A**) The reaction between Que and the Sirt3 protein. (**B**) Representative IF images of Sirt3 with different treatments in BMDMs. (**C**) Quantification analysis for Sirt3. (**D**) q-PCR assay of the gene expression of (i) Sirt3 and (ii) SOD2 genes. Data represent the mean ± SD; n = 3; ns: no significant; **** *p* < 0.0001.

The underling mechanism of Que in regulating macrophage polarization is still unclear. Previous reports indicated that Que plays a corresponding role by activating Sirt3 [32,33]. Sirt3, a member of the Sirtuin protein family, is an NAD+-dependent deacetylase located in mitochondria, which regulates various cellular processes such as energy metabolism, inflammation, and aging through the deacetylation modification of multiple proteins [34,35]. It has been found that Sirt3 can affect macrophage inflammatory responses by influencing the activation of NLRP3 inflammasomes [36]. Molecular docking analysis was used to investigate the binding between Que and Sirt3. AutoDock Vina v.1.2.2 showed the binding poses and observed the interactions between quercetin and Sirt3. The results revealed that Que forms visible hydrogen bonds and strong electrostatic interactions with the protein target of Sirt3. This information suggests a potential binding affinity between Que and Sirt3. The hydrophobic pocket of the target was successfully occupied by Que, and Que and Sirt3 had a low binding energy of −8.815 kcal/mol, indicating a good binding affinity between them (Figure 4A). Further experimental studies are needed to validate and elucidate the functional implications of this interaction.

SOD2, which is downstream from Sirt3, plays a significant role as an antioxidant enzyme in mitigating cellular damage and the progression of diseases triggered by inflammation [37,38]. However, it remains unclear whether QVR-CBMG could affect macrophage function via the Sirt3/SOD2 signaling pathway. Subsequently, further experiments were conducted to investigate the regulatory effect of QVR-CBMG on macrophage polarization. IF staining results showed that LPS led to a decrease in Sirt3 protein content, while QVR-CBMG effectively increased the expression of Sirt3 in macrophages (Figure 4B,C). QVR-CBMG treatment also increased the mRNA expression of Sirt3 and its downstream protein SOD2 (Figure 4D). In addition, the protein levels of Sirt3 and SOD2 in macrophages were detected via Western blot analysis, and the results revealed that an increase in the expression of Sirt3 and SOD2 was observed upon treatment with QVR-CBMG (Figure S3). These findings indicate that QVR-CBMG has the ability to stimulate the Sirt3/SOD2 signaling pathway in macrophages. Recent studies have reported that the repair process of diabetic wounds can be regulated by altering the expression of Sirt3 in macrophages [39]. Previous investigations have documented that a deficiency in Sirt3 can impede the healing process, impair blood supply, and downregulate the expression of vascular endothelial growth factor [40]. Conversely, activating Sirt3 has been demonstrated to effectively promote wound healing, aligning with our own findings [41]. Our study demonstrated that QVR-CBMG exerts its effects by activating the Sirt3/SOD2 signaling pathway. However, it is important to note that our research is limited by the lack of further exploration regarding the potential impact on the regulation of macrophage immune function when proteins implicated in the Sirt3-related pathway are inhibited. This direction will be a focus of our future investigations.

### 2.5. QVR-CBMG Hydrogel Accelerates Diabetic Infection Rat Wound Healing

**Figure 5:**
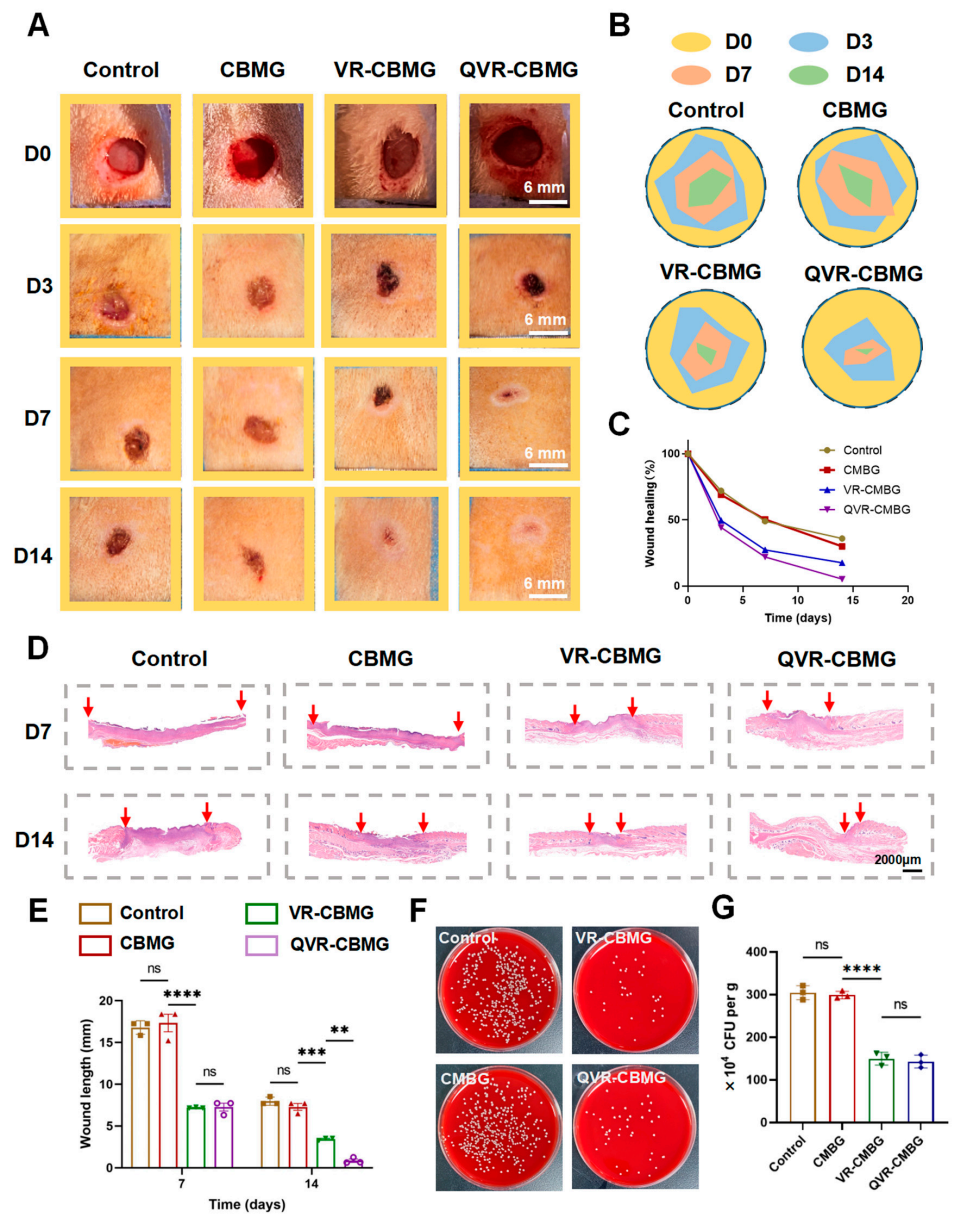
(**A**) Representative images of the wound-healing process in a diabetic infection model. (**B**) Schematic diagram of wound healing. (**C**) Quantitative data of the relative wound healing at different time points. (**D**) Representative images of wound tissue sections stained by H&E on D7 and D14. (**E**) Quantification of wound lengths in different groups. (**F**) Representative CFUs in wound tissues in different groups. (**G**) Analysis of the bacterial viability according to CFUs. Data represent the mean ± SD; n = 3; ns: no significant; ** *p* < 0.01, *** *p* < 0.001, and **** *p* < 0.0001.

The healing process of diabetic-infected wounds is complex and is impeded by various factors, such as drug-resistant bacterial infections, vascular impairment, oxidative damage, and immune microenvironment alterations [42,43]. To evaluate the potential of QVR-CBMG hydrogel in promoting wound healing, a diabetic wound infection rat model was established. Figure 5A presents the optical images of the wounds in each group using a digital camera, with simulated images at four time points shown in Figure 5B and the corresponding quantitative data analysis results in Figure 5C. These results indicate that the closure speed and rate of the wounds in the QVR-CBMG hydrogel group were faster than the other experimental groups. As shown in Figure 5D, the H&E staining results demonstrated that the QVR-CBMG group accelerated the wound healing compared to the other groups, which was consistent with the quantitative analysis of the wound healing outcomes on day 7 and day 14 (Figure 5E). 

The MRSA in infected skin tissues is represented in Figure 5B. Consistent with the in vitro antibacterial activity, the QVR-CBMG and VR-CBMG groups exhibited notably higher rates of bacterial clearance compared to the other groups. A bacterial presence, in particular, can produce toxins and release intracellular components, leading to tissue damage and necrosis at the wound site [44]. The remarkable effectiveness of QVR-CBMG and VR-CBMG hydrogels lies in their ability to effectively mitigate the persistent deterioration of wounds caused by bacterial biofilms. These hydrogels create a favorable local environment that facilitates wound healing by countering the detrimental effects of bacterial biofilms. In summary, these findings indicate that QVR-CBMG possesses a superior ability in promoting the healing of diabetic wounds. Applying QVR-CBMG to suppress bacterial infections in diabetic wounds has demonstrated a remarkable efficacy in promoting the healing process. Nevertheless, it is important to acknowledge that diabetic wounds primarily manifest in the lower extremities, particularly the feet [45]. Hence, future advancements should prioritize the development of rodent models with diabetic wounds specifically simulated in the lower limbs and feet, aiming to achieve an improved clinical relevance.

### 2.6. QVR-CBMG Hydrogel Promoted Collagen Repair and Angiogenesis In Vivo

**Figure 6:**
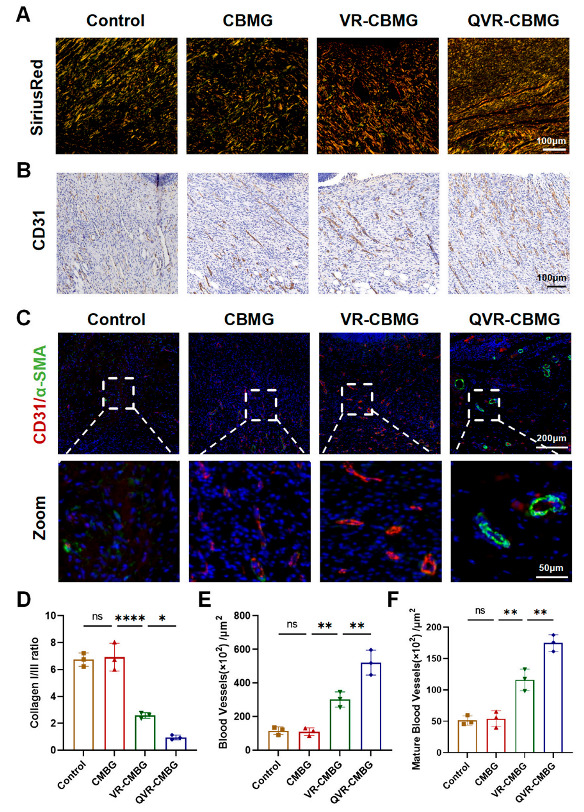
(**A**) Representative images of wound healing stained with Sirius red. (**B**) Representative IHC images of CD31 with different treatments. (**C**) Representative IF images of CD31/α-SMA with different treatments. (**D**) Analysis of the collagen I/III ratio in different groups. (**E**) Quantification of new blood vessels. (**F**) Quantification of mature blood vessels. Data represent the mean ± SD; n = 3; ns: no significant; * *p* < 0.05, ** *p* < 0.01 and **** *p* < 0.0001).

In the main structure of the skin, collagen fibers are mainly composed of thick collagen type I, which is a major component of skin tissue, and thin collagen type III, which is a major component of reticular fibers [46]. The normal ratio of collagen types I and III maintains the integrity of the skin tissue structure. The appropriate collagen ratio, development of new blood vessels, and adequate blood supply are essential for wound healing [47]. To investigate QVR-CBMG hydrogel in promoting collagen repair and angiogenesis, we observed the wound sites using polarized light imaging after Sirius Red staining. As shown in Figure 6A, the collagen I/III ratio in the QVR-CBMG treatment group was closer to 1, which is similar to normal skin [47,48]. This suggests that QVR-CBMG has a superior ability to promote collagen repair. Subsequently, we conducted IHC and IF staining to comprehensively evaluate the vascular components in each group. CD31 IHC staining (Figure 6B) and CD31/α-SMA dual IF staining (Figure 6C) demonstrated that the QVR-CBMG group exhibited significantly higher vascular density and quantity compared to the other groups. Quantitative analysis of the area of neovascularization and the number of neovessels (Figure 6E), as well as the total vessels and mature vessels (Figure 6F), yielded consistent results, indicating QVR-CBMG can facilitate vascularization in diabetic wounds. 

### 2.7. QVR-CBMG Hydrogel Facilitates M2 Polarization of Macrophages In Vivo

**Figure 7:**
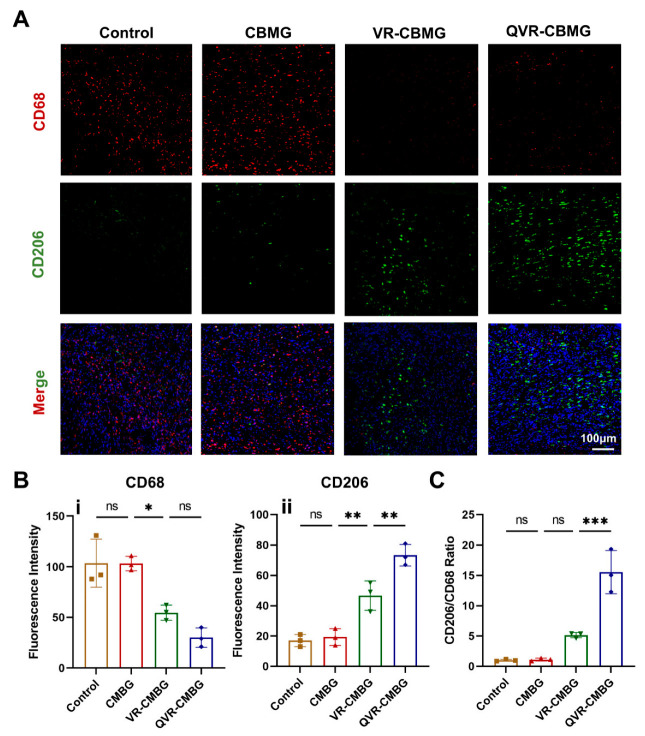
(**A**) Representative IF images of CD68/CD206 with different treatments. (**B**) Quantification analysis for CD68/CD206 (i,ii). (**C**) Analysis of the CD68/CD206 ratio in different groups. Data represent the mean ± SD; n = 3; ns: no significant; * *p* < 0.05, ** *p* < 0.01 and *** *p* < 0.001).

The immune microenvironment plays a crucial role in the process of diabetic wound healing [49,50]. CD86/CD206 serve as indicators for M1 and M2 macrophages, respectively, which are used to evaluate the changes in the immune microenvironment levels after different treatments in diabetic wound healing. The results revealed that, during the wound-healing process, the QVR-CBMG group exhibited the highest level of M2 macrophages (CD206) among the four groups, while the level of M1 macrophages (CD68) in the QVR-CBMG group was the lowest(Figure 7A). The corresponding statistical analysis and the CD68/CD206 ratio also supported these findings (Figure 7B,C), indicating that the QVR-CBMG treatment group experienced a faster transition into the prohealing stage of the immune microenvironment compared to the other groups, demonstrating its effective immune modulation function.

## 3. Conclusions

In conclusion, the QVR-CBMG hydrogel, which incorporates Rif, Van, and Que, has exhibited significant potential in enhancing the healing of diabetic wounds. The ease of preparation and sustained release of the loaded drugs make this multifunctional hydrogel highly advantageous. Moreover, the QVR-CBMG hydrogel effectively eliminates biofilms and provides comprehensive and long-lasting antibacterial protection for diabetic wounds. Additionally, this hydrogel promotes the polarization of macrophages towards the anti-inflammatory M2 phenotype by activating the Sirt3/SOD2 signaling pathway in macrophages. This mechanism creates a favorable microenvironment for tissue repair. Simultaneously, the hydrogel induces vascularization, enhances proliferation, promotes the formation of granulation tissue, and facilitates collagen accumulation, showcasing its immense potential in promoting diabetic skin regeneration. Overall, the QVR-CBMG hydrogel represents a multifunctional therapeutic approach capable of eliminating biofilm infection, fostering an anti-inflammatory microenvironment, and facilitating tissue regeneration.

## 4. Materials and Methods

### 4.1. Materials

Que, Van, and Rif were procured from Solarbio (Beijing, China). Carbomer 940 (powder, 99.0%) and triethanolamine (liquid, >99.5%) were obtained from Macklin Reagent Co. Ltd. (Shanghai, China). Methicillin-resistant *Staphylococcus aureus (*MRSA, ATCC43300) was a gift from the microbiology lab at the author’s institute.

### 4.2. Preparation of Hydrogels

Carbomer 940 was dissolved in ddH_2_O to prepare 1 wt% carbomer gel (CBMG) [21]. Specifically, 1 g of Carbomer 940 (Macklin Reagent, Shanghai, China) was dissolved in 100 mL of ddH_2_O and stirred at 60 °C for 30 min. Then, 0.25% triethanolamine (Adamas, Shanghai, China) was added into the carbomer solution to adjust the final pH to 6.5–7.5. After stirring for 10 min, CBMG was prepared. Afterwards, drugs (Rif, Van, and Que) in powders of different weights were mixed with CBMG under stirring for 30 min and left overnight at room temperature to form combined hydrogels. The crude hydrogels were washed three times with ddH_2_O to remove the uncombined material and stored in a sealed bottle at 4 °C.

### 4.3. Scanning Electron Microscope (SEM) Analysis

The microstructure of the hydrogel was analyzed via a SEM (SU-3500, HITACHI, Tokyo, Japan). As for the freeze-drying process, the hydrogels were frozen at −80 °C for 24 h and then subjected to freeze-drying at a pressure of 0.05 mbar for 24 h. Regarding the SEM imaging parameters, the voltage used was 5 kV, the current was 10 μA, and the detector used was SEM (SU-3500). 

### 4.4. Rheological Experiment

The hydrogel samples were prepared by mixing the drugs (Rif, Van, and Que) in a powdered form with the CBMG solution. The mixture was stirred for 30 min and left overnight at room temperature to form combined hydrogels. The resulting crude hydrogels were washed three times with ddH_2_O to remove the uncombined materials. The washed hydrogels were then stored in a sealed bottle at 4 °C.

To analyze the rheological properties of the hydrogels, rotary rheometer measurements were conducted. The hydrogel samples were placed between the rotor and the base of the rheometer to prevent slipping. A frequency of 1 Hz and a constant strain rate of 1% were maintained during the experiments. The measurements were performed within the linear viscoelastic range of the hydrogel. Three repetitions of the experiment were conducted, and a representative plot was selected to illustrate the changes in the elastic modulus, storage modulus, and the storage/loss modulus ratio of the hydrogel as a function of the temperature.

### 4.5. Cell Biocompatibilitye

CCK-8 kits (Solarbio, Beijing, China) were used to investigate the cell viability of BMDMs with samples at different concentrations. In brief, BMDMs were cultured with the control, CBMG, VR-CBMG, and QVR-CBMG for 1 day and 3 days, respectively. The cells were then washed and stained with 10% CCK-8 solution for 4 h. The relative cell density was measured using a microplate reader (BioTek Synergy Neo2, Winooski, VT, USA) at an absorbance value of 450 nm.

### 4.6. Macrophages Extraction and Culture

Primary rat bone marrow-derived macrophages (BMDMs) were obtained from Sprague–Dawley (SD) rats aged 28 days. Initially, bone marrow cells were extracted from the femur, followed by centrifugation at 450× *g* for 5 min. Subsequently, the cells were suspended in a red blood cell (RBC) lysis buffer for 15 min at 4 °C to purify the bone marrow cells. The suspended cells were then centrifuged at 500× *g* for 10 min to collect the BMDMs and eliminate the RBCs. Then, the cells were resuspended in 10% FBS/DMEM (high-glucose formulation) and cultured at 37 °C with 5% CO_2_. Nonadherent cells (monocytes) were collected and resuspended in an antibiotic-free complete medium containing 10% FBS and 15 ng/mL M-CSF after 48 h of culture. Macrophages were generated for 4 days in the presence of M-CSF.

### 4.7. Immunofluorescence (IF) Staining

IF staining was used to detect the protein levels of Sirt3 (1:400), CD68 (1:400), CD206 (1:400), CD31 (1:400), α-SMA (1:400), and F-actin (1:1000). Briefly, cells or tissues were immobilized with 4% paraformaldehyde for 15 min and then treated with 4% bovine serum albumin for 1 h. Afterward, the samples were incubated with antibodies overnight at 4 °C and labeled with secondary fluorophore antibodies (Servicebio, Wuhan, China) for 1.5 h. Nuclear staining was done using DAPI (Servicebio, China) for 15 min. Finally, all samples were observed via a confocal microscope (Olympus FV3000, Tokyo, Japan).

### 4.8. Western Blot (WB) Assay

RIPA lysis buffer (Beyotime, Shanghai, China) containing 1 mM phenylmethanesulfonylfluoride (BOSTER, Shenzhen, China) and phosphatase inhibitors (BOSTER, China) was used to extract cells. Following the determination of the protein concentrations using the BCA protein assay kit (Beyotime, China), 50 µg of protein extract was separated through gel electrophoresis and then transferred onto polyvinylidene fluoride membranes (Millipore, Burlington, MA, USA). After being blocked with StartingBlock (Thermo, Waltham, MA, USA) for 15 min, the membranes were incubated overnight at 4 °C with primary antibodies such as Sirt3 (1:1000), SOD2 (1:1000), and GAPDH (1:5000). Subsequently, the membranes underwent rinsing and were incubated for 2 h with horseradish peroxidase-labeled secondary antibodies (Solarbio, Beijing, China). Following rinsing, the protein signals were visualized using Image Lab 3.0 software.

### 4.9. Quantitative Polymerase Chain Reaction (qPCR)

The total RNAs were isolated from BMDMs following the provided guidelines (Accurate Biology, Hunan, China). The purity of the RNAs was assessed by measuring the OD at 260 and 280 nm, and a ratio of A260/A280 over 1.8 was considered satisfactory. The integrity of the RNAs was confirmed by agarose gel electrophoresis. cDNA synthesis was performed using the First-Strand Synthesis for qPCR kit (Accurate Biology, Hunan, China). RT-PCR was carried out using SYBR Premix Ex Taq kits (Accurate Biology, Hunan, China) and the Bio-Rad CFX96 Real-Time System (Bio-Rad, Hercules, CA, USA). Relative expression levels of the target genes were determined using the 2^−∆∆Ct^ method.

### 4.10. Antimicrobial Activity Tests In Vitro

The antibiofilm effect of QVR-CBMG was evaluated using crystal violet staining (Solarbio, China), the spread plate method, and Live/Dead bacterial staining (LIVE/DEAD BacLight Bacterial Viability Kit, Carlsbad, CA, USA). In brief, 100 µL of bacterial suspension (MRSA at 1 × 10^6^ CFU/mL) was added to 900 µL fresh TSB medium. After incubation for 48 h, the culture medium was removed, and the 48-h-old biofilm was treated with different hydrogels (10% in 1 mL fresh TSB) overnight. Then, the biofilm in each group was washed with PBS twice. 

The broth microdilution method was used to determine the minimum inhibitory concentration (MIC) for Van-CBMG. Firstly, 100 µL of diluted MRSA suspension (100×) was added to 10 wells in a 96-well plate, and then, 100 µL of Van-CBMG at a content of 512 μg/g was added to the first well. After two-fold serial dilution, the bacteria were grown overnight with different contents of Van-CBMG. Finally, the MIC was defined as the lowest Van-CBMG concentration at which no turbidity was observed in the corresponding well.

To determine the reciprocal reinforcement of Van combined with Rif to eradicate a mature biofilm, VR-CBMG with Van (4 μg/g) and various contents of Rif from 4 to 256 μg/g were used to treat a 48-h-old biofilm. Briefly, a 48-h-old biofilm was cultured in 1 mL fresh TSB medium with 50% VR-CBMG (*v*/*v*) overnight. The treated biofilm was rinsed, fixed, and then stained with 1% crystal violet.

For crystal violet staining, the biofilm was fixed with 4% glutaraldehyde for 20 min and then rinsed with PBS twice. Then, the biofilm was stained with 1% crystal violet for 5 min at room temperature. After washing with PBS twice, the optical images of crystal violet-stained biofilm were captured. After that, the samples were de-stained with 30% acetic acid solution and semi-quantitatively measured using a microplate reader at 570 nm.

For the quantitative analysis of antibiofilm activity, the biofilm was detached by ultrasonic oscillation. Then, 100 µL of serially diluted bacterial suspension was plated onto sheep blood agar plates and incubated overnight. The antibiofilm rate was calculated using the formula (CFUs in control)−(CFUs in hydrogel)/CFUs in control × 100%.The treated biofilm was stained with 0.1% Styo9 and propidium iodide (PI) for 15 min in the dark. Then, the samples were rinsed with PBS twice before confocal observation. The data were presented as a live/dead ratio, which was measured by ImageJ (V1.8.0, NIH, Bethesda, MD, USA).

### 4.11. Rats Model and Treatment

Female Sprague–Dawley (SD) rats weighing 200 g were obtained from the Laboratory of Animal Center (The First Affiliated Hospital of Zhejiang University). These rats were housed in a specific pathogen-free (SPF) environment at a temperature of 22 ± 1 °C with a 12-h light–dark cycle. Diabetic animal models were induced by administering streptozotocin in a citric acid buffer (50 mg/kg). Fasting blood glucose levels above 16.67 mmol/L after one week were considered a successful model. Then, the rats were anesthetized with an intraperitoneal administration of pentobarbital (40 mg/kg). After shaving and disinfection, circular full-thickness skin defects with a diameter of 10.0 mm were created on the dorsal side of each rat. Next, each defect area was infected with 10.0 µL of MRSA suspension (1 × 10^8^ CFU/mL). The wound sites were then treated with approximately 200 µL of the hydrogel at 48 h post-infection. The optical images of the wound were captured using a digital camera on days 0, 3, 7, and 14, respectively. Five rats from each group were randomly selected for bacteriological assessments on day 3.

### 4.12. Histological and Immunostaining Analyses

For the histological and immunostaining analyses, the rats from each group were euthanized 14 days after treatment. The wound specimens were collected and fixed in 4% paraformaldehyde for 48 h. Slices 5 µm thick were prepared for hematoxylin and eosin (H&E) staining and Sirius red staining (polarized light). Immunohistochemical staining was performed using antibodies against CD31 (1:400) and α-SMA (1:400) to investigate angiogenesis during the wound-healing process. Additionally, CD68 (1:400)/CD206 (1:400) antibodies were utilized to identify polarized macrophages.

### 4.13. Statistical Analysis

Each experiment was performed at least three times. One-way analysis of variance (ANOVA) followed by Tukey’s post hoc test were conducted using SPSS 26 (SPSS Inc., Chicago, IL, USA). All data are shown as the mean ± standard deviation (SD), and a *p*-value less than 0.05 was considered statistically significant.

## Data Availability

The datasets utilized and analyzed in this article can be obtained from the corresponding author upon reasonable request.

## Supplementary Materials

The following supporting information can be downloaded at: https://www.mdpi.com/article/10.3390/gels9090761/s1, Figure S1: A. The CCK-8 results of macrophages on 1 days. B. The CCK-8 results of macrophages on 3 days. OD, optical density. (Data represen mean ± SD, n = 3, * *p* < 0.05, ** *p* < 0.01, *** *p* < 0.001 and **** *p*< 0.0001); Figure S2: A. The broth microdilution method to determine the minimum inhibitory concentration of Van-CBMG against MRSA. B. Crystal violet staining of biofilm after treatment with different samples. The composition of VR-CBMG was mixed with Van (4 μg/g) and various contents of Rif from 4 μg/g -256 μg/g; Figure S3: A. Western blot of Sirt3/SOD2 in different groups. B. Protein expression result of Sirt3/SOD2. (Data represen mean ± SD, n = 3, * *p* < 0.05, ** *p* < 0.01, *** *p* < 0.001 and **** *p*< 0.0001).
