# Supplementary material for: Multifunctional Drugs-Loaded Carbomol Hydrogel Promotes Diabetic Wound Healing via Antimicrobial and Immunoregulation
Source: Gels. 2023 Sep 18;9(9):761. doi: 10.3390/gels9090761 (PMC10530860; doi:10.3390/gels9090761)
Supplement: Supplementary file 1 [file gels-09-00761-s001.zip › gels-2572082-supplementary.pdf]

Multifunctional Drugs-Loaded Carbomol Hydrogel Promotes Diabetic Wound Healing via Antimicrobial and Immunoregulation

Hehui Wang [1,2,†], Jiale Jin [2,†], Chi Zhang [1], Fangyi Gong [1], Baiwen Hu [1], Xiaochuan Wu [1], Ming Guan [2,*] and Dongdong Xia [1,*]

[1] Department of Orthopedics, The First Affiliated Hospital of Ningbo University, Ningbo 315000, China; fyywanghehui@nbu.edu.cn (H.W.); fyyzhangchi@nbu.edu.cn (C.Z.); fyygongfangyi@nbu.edu.cn (F.G.); fyyhubaiwen@nbu.edu.cn (B.H.); fyywuxiaochuan@nbu.edu.cn (X.W.)

[2] Department of Orthopedics, The First Affiliated Hospital, Zhejiang University School of Medicine, Hangzhou 310000, China; 22018143@zju.edu.cn

* Correspondence: guanm@zju.edu.cn (M.G.); fyyxiadongdong@nbu.edu.cn (D.X.)

† These authors contributed equally to this work.

1. Supporting figures

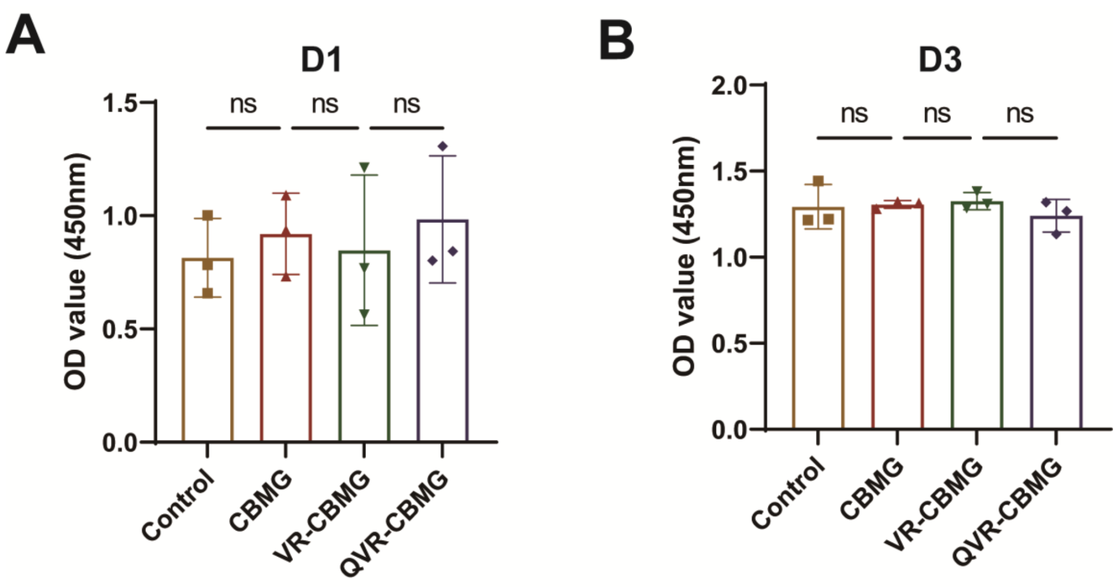

Figure S1. A. The CCK-8 results of macrophages on 1 days. **B.** The CCK-8 results of macrophages on 3 days. OD, optical density. (Data represen mean ± SD, n = 3, $*p < 0.05$, $**p < 0.01$, $***p < 0.001$ and $****p < 0.0001$).

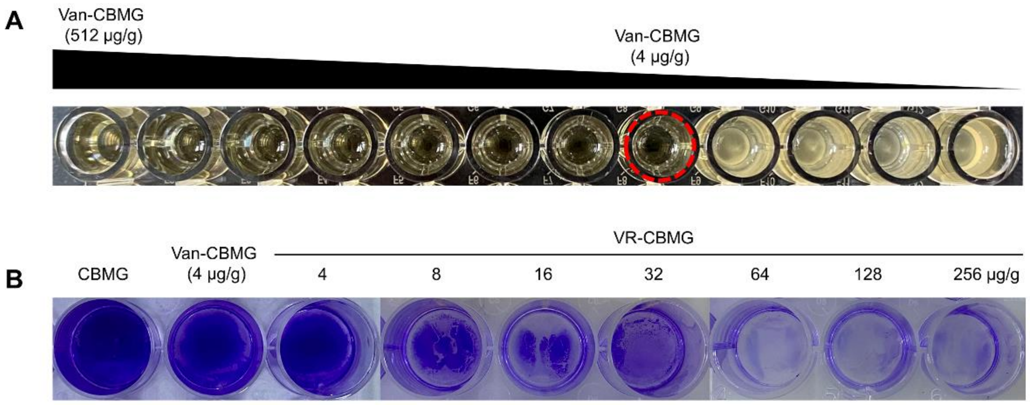

Figure S2. A. The broth microdilution method to determine the minimum inhibitory concentration of Van-CBMG against MRSA. **B.** Crystal violet staining of biofilm after treatment with different samples. The composition of VR-CBMG was mixed with Van (4 μg/g) and various contents of Rif from 4 μg/g -256 μg/g.

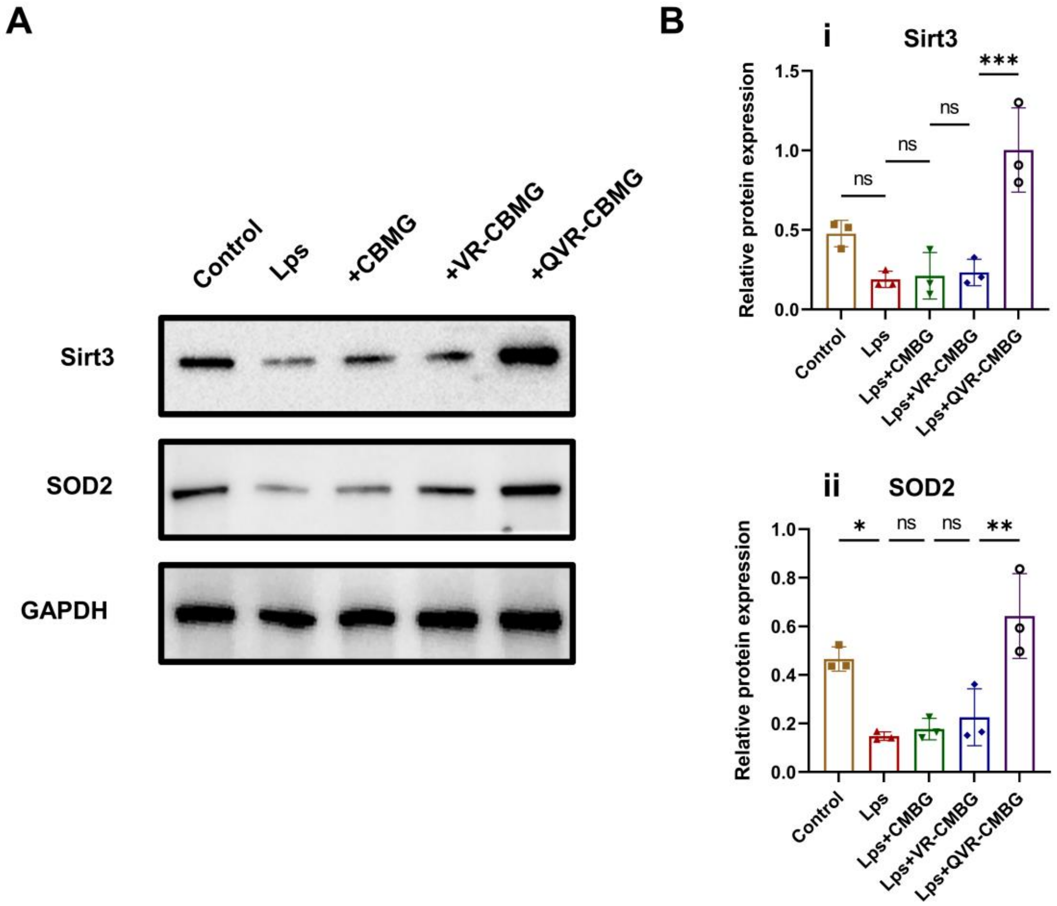

Figure S3. A. Western blot of Sirt3/SOD2 in different groups. **B.** Protein expression result of Sirt3/SOD2. (Data represen mean ± SD, n = 3, *p < 0.05, **p < 0.01, ***p < 0.001 and ****p< 0.0001).
